# Bis(benzyl­trimethyl­ammonium) tetra­bromidocuprate(II)

**DOI:** 10.1107/S1600536811034830

**Published:** 2011-08-31

**Authors:** Lei Jin, Ning Liu, Yong-Jun Li, De-Hong Wu

**Affiliations:** a College of Chemistry and Chemical Engineering, Southeast University, Nanjing 210096, People’s Republic of China

## Abstract

In the title mol­ecular salt, (C_10_H_16_N)_2_[CuBr_4_], the Cu^II^ ion adopts a squashed tetra­hedral geometry with Br—Cu—Br angles varying between 99.29 (3) and 132.53 (3)°. In the crystal, the components are linked by C—H⋯Br inter­actions, thereby generating a three-dimensional network.

## Related literature

For background to mol­ecular–ionic compounds, see: Coffey *et al.* (2000 ▶); Liu *et al.* (2001 ▶); Long *et al.* (1997 ▶); Luque *et al.* (1997 ▶); Woodward *et al.* (2001 ▶).
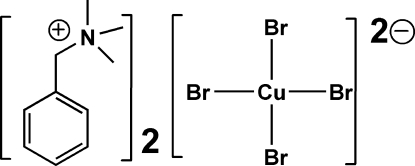


## Experimental

### 

#### Crystal data

(C_10_H_16_N)_2_[CuBr_4_]
*M* *_r_* = 683.66
Orthorhombic, 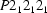

*a* = 9.1908 (8) Å
*b* = 9.6697 (19) Å
*c* = 29.0243 (8) Å
*V* = 2579.5 (6) Å^3^
*Z* = 4
Mo *K*α radiation
μ = 7.05 mm^−1^
*T* = 291 K
0.28 × 0.26 × 0.24 mm

#### Data collection

Rigaku Mercury2 diffractometer
Absorption correction: multi-scan (*CrystalClear*; Rigaku, 2005 ▶) *T*_min_ = 0.161, *T*_max_ = 0.183
25511 measured reflections
5922 independent reflections
3769 reflections with *I* > 2σ(*I*)
*R*_int_ = 0.101

#### Refinement

*R*[*F* ^2^ > 2σ(*F* ^2^)] = 0.063
*wR*(*F* ^2^) = 0.172
*S* = 1.02
5922 reflections
250 parameters
H-atom parameters constrained
Δρ_max_ = 0.89 e Å^−3^
Δρ_min_ = −1.33 e Å^−3^
Absolute structure: Flack (1983 ▶), 2556 Friedel pairs
Flack parameter: 0.06 (2)

### 

Data collection: *CrystalClear* (Rigaku, 2005 ▶); cell refinement: *CrystalClear*; data reduction: *CrystalClear*; program(s) used to solve structure: *SHELXS97* (Sheldrick, 2008 ▶); program(s) used to refine structure: *SHELXL97* (Sheldrick, 2008 ▶); molecular graphics: *SHELXTL* (Sheldrick, 2008 ▶); software used to prepare material for publication: *SHELXTL*.

## Supplementary Material

Crystal structure: contains datablock(s) I, global. DOI: 10.1107/S1600536811034830/hb6385sup1.cif

Structure factors: contains datablock(s) I. DOI: 10.1107/S1600536811034830/hb6385Isup2.hkl

Additional supplementary materials:  crystallographic information; 3D view; checkCIF report

## Figures and Tables

**Table 1:** Selected bond lengths (Å)

Cu1—Br1	2.3522 (7)
Cu1—Br2	2.3912 (7)
Cu1—Br3	2.3764 (7)
Cu1—Br4	2.3378 (7)

**Table 2:** Hydrogen-bond geometry (Å, °)

*D*—H⋯*A*	*D*—H	H⋯*A*	*D*⋯*A*	*D*—H⋯*A*
C8—H8⋯Br3^i^	0.93	2.90	3.737 (5)	150
C12—H12*A*⋯Br4^ii^	0.96	2.89	3.781 (5)	155
C12—H12*C*⋯Br4^iii^	0.96	2.93	3.794 (5)	151

Symmetry codes: (i) 
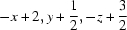
; (ii) 
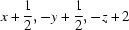
; (iii) 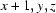.

**Fig. 1.**
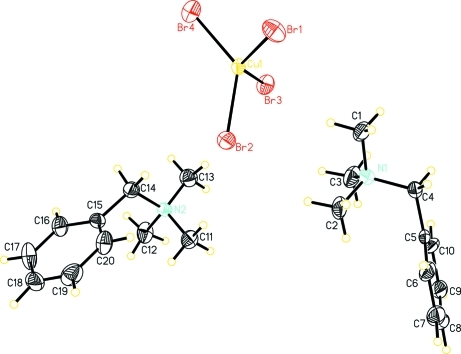
The molecular structure of the title compound showing 30% probability displacement ellipsoids.

## supplementary crystallographic information

### Comment

Recently much attention has been devoted to simple molecular–ionic compounds containing organic cations and anions due to the tunability of their special structural features and their interesting physical properties (Coffey *et al.*, 2000; Liu *et al.*, 2001; Long *et al.*, 1997; Luque *et al.*, 1997; Woodward *et al.*, 2001). In our laboratory, the title compound has been synthesized and its crystal structure is herein reported.

The molecule of the title compound, (C_10_H_16_N^+^)_2_.CuBr_4_^2-^crystallizes in theorthorhombic *P*2_1_2_1_2_1_ space group, and an asymmetric unit consists of one bromocuprate anion unitand two benzyltrimethylammonium cations (Fig 1). In the structure, the Cu(II) ion adopts a distorted tetrahedron geometry by four Br^-^ anions with the bond distances of Cd–Br being in the range of 2.3378 (7)–2.3912 (7) Å and the bond angles of Br–Cd–Br being in the range of 99.29 (3)–132.53 (3)°, thus largely deviating from ideal tetrahedral angles of 109.5°. There are no classic hydrogen bonds found except for nonclassic C(8)—H(8)···Br(3), C(12)—H(12 A)···Br(34), C(12)—H(12 C)···Br(4) hydrogen-bonded interactions (Table 1). The benzyltrimethylammonium cations interact with the tetrahedral CuBr_4_^2-^ anionthrough above nonclassic hydrogen-bonded interactionsand non-covalent interaction-static attracting forces (Coulomb and Van der Waals forces) to afford a three-dimensional network.

### Experimental

At room temperature, benzyltrimethylammoniumchlorine (5 mmol, 0.93 g) were dissolved in 30 ml ethanol, then CuCl_2_^.^H_2_O (5 mmol, 0.85 g) was added into the previous solution slowly with sirring. A great quantity of yellow microcrystasls were obtained by filtrating after 3 days in air. The crystal was further dissolved in ethanol with excessive HBr solution carefully added with stiring. A purple solid appeared after days in the air with yield about 65%. Block purple single crystals were obtained by the slow evaporation of the above solution after a week in air.

The dielectric constant of the compound as a function of temperature indicates that the permittivity is basically temperature-independent (ε = C/(T–T_0_)), suggesting that this compound is not ferroelectric or there may be no distinct phase transition occurring within the measured temperature range between 123 K and 400 K (below the compound melting point 420 K).

### Refinement

H atoms were placed in calculated positions(C—H = 0.93Å for C*sp^2^* atoms and C—H = 0.96Å and 0.96Å for C*sp^3^* atoms), assigned fixed *U*_iso_ values [*U*_iso_ = 1.2*U*eq(C*sp^2^*/N) and 1.5*U*eq(C*sp^3^*)] and allowed to ride.

### Crystal data

**Table:**

(C_10_H_16_N)_2_[CuBr_4_]	*F*(000) = 1340
*M**_r_* = 683.66	*D*_x_ = 1.760 Mg m^−^^3^
Orthorhombic, *P*2_1_2_1_2_1_	Mo *K*α radiation, λ = 0.71073 Å
Hall symbol: P 2ac 2ab	Cell parameters from 20422 reflections
*a* = 9.1908 (8) Å	θ = 3.1–27.8°
*b* = 9.6697 (19) Å	µ = 7.05 mm^−^^1^
*c* = 29.0243 (8) Å	*T* = 291 K
*V* = 2579.5 (6) Å^3^	Block, purple
*Z* = 4	0.28 × 0.26 × 0.24 mm

### Data collection

**Table:**

Rigaku Mercury2 diffractometer	5922 independent reflections
Radiation source: fine-focus sealed tube	3769 reflections with *I* > 2σ(*I*)
graphite	*R*_int_ = 0.101
Detector resolution: 13.6612 pixels mm^-1^	θ_max_ = 27.5°, θ_min_ = 3.1°
CCD_Profile_fitting scans	*h* = −11→11
Absorption correction: multi-scan (*CrystalClear*; Rigaku, 2005)	*k* = −12→12
*T*_min_ = 0.161, *T*_max_ = 0.183	*l* = −37→37
25511 measured reflections	

### Refinement

**Table:**

Refinement on *F*^2^	Secondary atom site location: difference Fourier map
Least-squares matrix: full	Hydrogen site location: inferred from neighbouring sites
*R*[*F*^2^ > 2σ(*F*^2^)] = 0.063	H-atom parameters constrained
*wR*(*F*^2^) = 0.172	*w* = 1/[σ^2^(*F*_o_^2^) + (0.0838*P*)^2^] where *P* = (*F*_o_^2^ + 2*F*_c_^2^)/3
*S* = 1.02	(Δ/σ)_max_ < 0.001
5922 reflections	Δρ_max_ = 0.89 e Å^−^^3^
250 parameters	Δρ_min_ = −1.33 e Å^−^^3^
0 restraints	Absolute structure: Flack (1983), 2556 Friedel pairs
Primary atom site location: structure-invariant direct methods	Flack parameter: 0.06 (2)

### Special details

**Table:**

Geometry. All e.s.d.'s (except the e.s.d. in the dihedral angle between two l.s. planes) are estimated using the full covariance matrix. The cell e.s.d.'s are taken into account individually in the estimation of e.s.d.'s in distances, angles and torsion angles; correlations between e.s.d.'s in cell parameters are only used when they are defined by crystal symmetry. An approximate (isotropic) treatment of cell e.s.d.'s is used for estimating e.s.d.'s involving l.s. planes.
Refinement. Refinement of *F*^2^ against ALL reflections. The weighted *R*-factor *wR* and goodness of fit *S* are based on *F*^2^, conventional *R*-factors *R* are based on *F*, with *F* set to zero for negative *F*^2^. The threshold expression of *F*^2^ > σ(*F*^2^) is used only for calculating *R*-factors(gt) *etc*. and is not relevant to the choice of reflections for refinement. *R*-factors based on *F*^2^ are statistically about twice as large as those based on *F*, and *R*- factors based on ALL data will be even larger.

### Fractional atomic coordinates and isotropic or equivalent isotropic displacement parameters (Å^2^)

**Table:**

	*x*	*y*	*z*	*U*_iso_*/*U*_eq_	
Br1	0.16703 (6)	0.33426 (5)	0.800673 (18)	0.06775 (16)	
Br2	0.49529 (5)	0.46102 (5)	0.855426 (17)	0.05492 (13)	
Br3	0.48651 (5)	0.08709 (5)	0.870934 (18)	0.05845 (14)	
Br4	0.15255 (6)	0.21832 (6)	0.91811 (2)	0.07334 (17)	
C1	0.5435 (5)	0.2722 (7)	0.73255 (16)	0.077 (2)	
H1A	0.4986	0.3359	0.7115	0.115*	
H1B	0.5080	0.2889	0.7631	0.115*	
H1C	0.5203	0.1792	0.7236	0.115*	
C2	0.7333 (6)	0.4347 (5)	0.74409 (16)	0.0749 (18)	
H2A	0.6761	0.4959	0.7254	0.112*	
H2B	0.8348	0.4530	0.7391	0.112*	
H2C	0.7100	0.4493	0.7760	0.112*	
C3	0.7683 (5)	0.1951 (6)	0.76459 (15)	0.0680 (17)	
H3A	0.7392	0.2194	0.7953	0.102*	
H3B	0.8723	0.2005	0.7621	0.102*	
H3C	0.7371	0.1025	0.7579	0.102*	
C4	0.7539 (4)	0.2631 (4)	0.68414 (13)	0.0441 (12)	
H4A	0.6950	0.3158	0.6627	0.053*	
H4B	0.7387	0.1659	0.6775	0.053*	
C5	0.9108 (4)	0.2968 (4)	0.67551 (13)	0.0351 (11)	
C6	0.9473 (5)	0.4283 (5)	0.65858 (15)	0.0537 (14)	
H6	0.8759	0.4939	0.6524	0.064*	
C7	1.0959 (6)	0.4577 (5)	0.65128 (17)	0.0684 (17)	
H7	1.1235	0.5444	0.6405	0.082*	
C8	1.1977 (5)	0.3617 (6)	0.65970 (15)	0.0683 (17)	
H8	1.2945	0.3852	0.6547	0.082*	
C9	1.1682 (5)	0.2327 (5)	0.67503 (15)	0.0569 (14)	
H9	1.2420	0.1687	0.6803	0.068*	
C10	1.0156 (4)	0.1981 (4)	0.68298 (14)	0.0474 (12)	
H10	0.9897	0.1101	0.6930	0.057*	
C11	0.8707 (5)	0.5105 (5)	0.91952 (14)	0.0533 (14)	
H11A	0.9518	0.4566	0.9089	0.080*	
H11B	0.9034	0.6016	0.9276	0.080*	
H11C	0.7992	0.5168	0.8955	0.080*	
C12	0.9205 (5)	0.4376 (6)	0.99779 (15)	0.0609 (16)	
H12A	0.8770	0.4072	1.0261	0.091*	
H12B	0.9617	0.5279	1.0020	0.091*	
H12C	0.9956	0.3740	0.9889	0.091*	
C13	0.7555 (5)	0.3076 (5)	0.95072 (16)	0.0596 (16)	
H13A	0.6932	0.3096	0.9242	0.089*	
H13B	0.7022	0.2728	0.9767	0.089*	
H13C	0.8372	0.2485	0.9447	0.089*	
C14	0.6765 (5)	0.5276 (4)	0.97618 (16)	0.0482 (13)	
H14A	0.6285	0.4785	1.0010	0.058*	
H14B	0.6080	0.5347	0.9509	0.058*	
C15	0.7132 (4)	0.6732 (4)	0.99279 (15)	0.0426 (12)	
C16	0.7364 (5)	0.6983 (5)	1.03985 (15)	0.0567 (15)	
H16	0.7287	0.6280	1.0616	0.068*	
C17	0.7715 (6)	0.8337 (6)	1.05265 (17)	0.0758 (18)	
H17	0.7846	0.8544	1.0837	0.091*	
C18	0.7868 (5)	0.9352 (5)	1.0209 (2)	0.0659 (17)	
H18	0.8184	1.0222	1.0302	0.079*	
C19	0.7564 (5)	0.9120 (6)	0.9752 (2)	0.0732 (18)	
H19	0.7613	0.9844	0.9542	0.088*	
C20	0.7181 (5)	0.7791 (5)	0.96046 (16)	0.0584 (15)	
H20	0.6963	0.7620	0.9297	0.070*	
Cu1	0.32377 (5)	0.27605 (5)	0.861735 (16)	0.03655 (13)	
N1	0.7012 (3)	0.2913 (3)	0.73160 (12)	0.0441 (10)	
N2	0.8055 (3)	0.4434 (3)	0.96042 (12)	0.0401 (10)	

### Atomic displacement parameters (Å^2^)

**Table:**

	*U*^11^	*U*^22^	*U*^33^	*U*^12^	*U*^13^	*U*^23^
Br1	0.0715 (3)	0.0703 (3)	0.0614 (3)	0.0008 (3)	−0.0305 (3)	0.0075 (3)
Br2	0.0561 (2)	0.0591 (3)	0.0496 (3)	−0.0083 (2)	0.0004 (2)	−0.0010 (2)
Br3	0.0548 (2)	0.0547 (2)	0.0659 (3)	0.0217 (2)	0.0048 (2)	−0.0025 (2)
Br4	0.0754 (3)	0.0813 (3)	0.0634 (3)	0.0056 (3)	0.0165 (3)	−0.0031 (3)
C1	0.040 (2)	0.150 (5)	0.040 (3)	−0.008 (3)	0.004 (2)	−0.014 (3)
C2	0.108 (4)	0.060 (3)	0.057 (3)	−0.015 (3)	0.033 (3)	−0.026 (2)
C3	0.068 (3)	0.096 (4)	0.040 (3)	0.007 (3)	0.008 (3)	0.006 (3)
C4	0.0396 (19)	0.054 (2)	0.039 (2)	−0.002 (2)	0.0012 (19)	−0.012 (2)
C5	0.0446 (19)	0.036 (2)	0.025 (2)	−0.0101 (18)	0.0075 (17)	−0.0088 (17)
C6	0.069 (3)	0.054 (3)	0.038 (3)	−0.006 (2)	0.015 (2)	0.004 (2)
C7	0.104 (4)	0.052 (3)	0.050 (3)	−0.021 (3)	0.016 (3)	0.004 (2)
C8	0.056 (3)	0.109 (4)	0.040 (3)	−0.035 (3)	0.010 (2)	−0.009 (3)
C9	0.053 (2)	0.082 (3)	0.037 (3)	−0.007 (3)	−0.005 (2)	0.000 (2)
C10	0.043 (2)	0.053 (2)	0.047 (2)	−0.004 (2)	0.019 (2)	−0.003 (2)
C11	0.070 (3)	0.056 (2)	0.034 (2)	−0.005 (2)	0.003 (2)	0.012 (2)
C12	0.059 (3)	0.089 (3)	0.035 (3)	0.021 (3)	−0.017 (2)	−0.003 (3)
C13	0.061 (3)	0.053 (3)	0.065 (3)	0.001 (2)	0.000 (3)	−0.002 (2)
C14	0.041 (2)	0.059 (3)	0.045 (3)	−0.002 (2)	−0.002 (2)	−0.004 (2)
C15	0.036 (2)	0.043 (2)	0.048 (3)	−0.0017 (19)	−0.0012 (19)	−0.005 (2)
C16	0.070 (3)	0.062 (3)	0.038 (3)	0.003 (3)	−0.002 (2)	−0.004 (2)
C17	0.107 (4)	0.074 (3)	0.046 (3)	0.024 (3)	−0.010 (3)	−0.021 (3)
C18	0.056 (3)	0.045 (3)	0.096 (4)	0.001 (2)	0.006 (3)	−0.020 (3)
C19	0.084 (3)	0.061 (3)	0.075 (4)	0.005 (3)	0.024 (3)	0.017 (3)
C20	0.078 (3)	0.056 (3)	0.041 (3)	0.020 (3)	0.003 (2)	0.001 (2)
Cu1	0.0384 (2)	0.0409 (2)	0.0304 (3)	0.0049 (2)	−0.0006 (2)	−0.0023 (2)
N1	0.0378 (17)	0.0514 (19)	0.043 (2)	−0.0065 (17)	0.0017 (15)	−0.0093 (17)
N2	0.0329 (16)	0.0449 (19)	0.042 (2)	−0.0031 (16)	−0.0029 (15)	−0.0020 (16)

### Geometric parameters (Å, °)

**Table:**

Cu1—Br1	2.3522 (7)	C9—H9	0.9300
Cu1—Br2	2.3912 (7)	C10—H10	0.9300
Cu1—Br3	2.3764 (7)	C11—N2	1.480 (5)
Cu1—Br4	2.3378 (7)	C11—H11A	0.9600
C1—N1	1.461 (5)	C11—H11B	0.9600
C1—H1A	0.9600	C11—H11C	0.9600
C1—H1B	0.9600	C12—N2	1.515 (5)
C1—H1C	0.9600	C12—H12A	0.9600
C2—N1	1.463 (6)	C12—H12B	0.9600
C2—H2A	0.9600	C12—H12C	0.9600
C2—H2B	0.9600	C13—N2	1.420 (6)
C2—H2C	0.9600	C13—H13A	0.9600
C3—N1	1.471 (6)	C13—H13B	0.9600
C3—H3A	0.9600	C13—H13C	0.9600
C3—H3B	0.9600	C14—N2	1.509 (5)
C3—H3C	0.9600	C14—C15	1.525 (6)
C4—N1	1.485 (5)	C14—H14A	0.9700
C4—C5	1.499 (5)	C14—H14B	0.9700
C4—H4A	0.9700	C15—C20	1.390 (6)
C4—H4B	0.9700	C15—C16	1.403 (6)
C5—C10	1.374 (5)	C16—C17	1.398 (7)
C5—C6	1.404 (6)	C16—H16	0.9300
C6—C7	1.411 (7)	C17—C18	1.353 (7)
C6—H6	0.9300	C17—H17	0.9300
C7—C8	1.340 (7)	C18—C19	1.373 (8)
C7—H7	0.9300	C18—H18	0.9300
C8—C9	1.352 (7)	C19—C20	1.400 (7)
C8—H8	0.9300	C19—H19	0.9300
C9—C10	1.460 (6)	C20—H20	0.9300
			
Br4—Cu1—Br1	99.92 (3)	N2—C11—H11C	109.5
Br4—Cu1—Br3	99.29 (3)	H11A—C11—H11C	109.5
Br1—Cu1—Br3	130.85 (3)	H11B—C11—H11C	109.5
Br4—Cu1—Br2	132.53 (3)	N2—C12—H12A	109.5
Br1—Cu1—Br2	99.62 (3)	N2—C12—H12B	109.5
Br3—Cu1—Br2	99.72 (3)	H12A—C12—H12B	109.5
N1—C1—H1A	109.5	N2—C12—H12C	109.5
N1—C1—H1B	109.5	H12A—C12—H12C	109.5
H1A—C1—H1B	109.5	H12B—C12—H12C	109.5
N1—C1—H1C	109.5	N2—C13—H13A	109.5
H1A—C1—H1C	109.5	N2—C13—H13B	109.5
H1B—C1—H1C	109.5	H13A—C13—H13B	109.5
N1—C2—H2A	109.5	N2—C13—H13C	109.5
N1—C2—H2B	109.5	H13A—C13—H13C	109.5
H2A—C2—H2B	109.5	H13B—C13—H13C	109.5
N1—C2—H2C	109.5	N2—C14—C15	114.8 (3)
H2A—C2—H2C	109.5	N2—C14—H14A	108.6
H2B—C2—H2C	109.5	C15—C14—H14A	108.6
N1—C3—H3A	109.5	N2—C14—H14B	108.6
N1—C3—H3B	109.5	C15—C14—H14B	108.6
H3A—C3—H3B	109.5	H14A—C14—H14B	107.5
N1—C3—H3C	109.5	C20—C15—C16	121.6 (4)
H3A—C3—H3C	109.5	C20—C15—C14	118.3 (4)
H3B—C3—H3C	109.5	C16—C15—C14	120.1 (4)
N1—C4—C5	115.4 (3)	C17—C16—C15	117.1 (4)
N1—C4—H4A	108.4	C17—C16—H16	121.4
C5—C4—H4A	108.4	C15—C16—H16	121.4
N1—C4—H4B	108.4	C18—C17—C16	121.5 (5)
C5—C4—H4B	108.4	C18—C17—H17	119.3
H4A—C4—H4B	107.5	C16—C17—H17	119.3
C10—C5—C6	121.1 (4)	C17—C18—C19	121.2 (5)
C10—C5—C4	119.8 (4)	C17—C18—H18	119.4
C6—C5—C4	119.0 (4)	C19—C18—H18	119.4
C5—C6—C7	117.8 (4)	C18—C19—C20	119.8 (5)
C5—C6—H6	121.1	C18—C19—H19	120.1
C7—C6—H6	121.1	C20—C19—H19	120.1
C8—C7—C6	120.6 (5)	C15—C20—C19	118.5 (4)
C8—C7—H7	119.7	C15—C20—H20	120.7
C6—C7—H7	119.7	C19—C20—H20	120.7
C7—C8—C9	124.1 (5)	C1—N1—C2	108.4 (4)
C7—C8—H8	118.0	C1—N1—C3	108.9 (4)
C9—C8—H8	118.0	C2—N1—C3	110.7 (4)
C8—C9—C10	117.1 (4)	C1—N1—C4	108.6 (3)
C8—C9—H9	121.4	C2—N1—C4	109.7 (3)
C10—C9—H9	121.4	C3—N1—C4	110.5 (3)
C5—C10—C9	119.3 (4)	C13—N2—C11	112.2 (3)
C5—C10—H10	120.3	C13—N2—C14	107.8 (3)
C9—C10—H10	120.3	C11—N2—C14	108.9 (3)
N2—C11—H11A	109.5	C13—N2—C12	109.5 (3)
N2—C11—H11B	109.5	C11—N2—C12	107.9 (3)
H11A—C11—H11B	109.5	C14—N2—C12	110.5 (3)

### Hydrogen-bond geometry (Å, °)

**Table:**

*D*—H···*A*	*D*—H	H···*A*	*D*···*A*	*D*—H···*A*
C8—H8···Br3^i^	0.93	2.90	3.737 (5)	150
C12—H12A···Br4^ii^	0.96	2.89	3.781 (5)	155
C12—H12C···Br4^iii^	0.96	2.93	3.794 (5)	151

Symmetry codes: (i) −*x*+2, *y*+1/2, −*z*+3/2; (ii) *x*+1/2, −*y*+1/2, −*z*+2; (iii) *x*+1, *y*, *z*.
